# From Cervical Trauma and Head Injury to Malignant Cerebral Edema: Blunt Cerebrovascular Injury Causing Large-Vessel Occlusion Treated With Thrombectomy and Neurocritical Care

**DOI:** 10.7759/cureus.106001

**Published:** 2026-03-27

**Authors:** Shunsuke Hatakenaka, Tatsuya Tanaka, Eiichi Suehiro, Akira Matsuno

**Affiliations:** 1 Department of Neurosurgery, International University of Health and Welfare Narita Hospital, Narita, JPN

**Keywords:** blunt cerebrovascular injury, intracranial pressure monitoring, large vessel occlusion, mechanical thrombectomy, temperature management, traumatic carotid artery dissection

## Abstract

Blunt cerebrovascular injury (BCVI) is an uncommon but potentially life-threatening complication of cervical trauma that may lead to delayed ischemic stroke. Early diagnosis is often challenging because neurological symptoms may initially be absent or mild. We report a case of traumatic internal carotid artery dissection associated with cervical fracture that resulted in large-vessel occlusion and required both endovascular treatment and intensive neurocritical care. A 57-year-old woman fell down stairs and sustained cervical trauma. Initial computed tomography revealed a fracture of the atlas involving the transverse foramen without intracranial hemorrhage. Approximately four hours after the injury, she developed right hemiparesis, aphasia, and left conjugate deviation. Computed tomography angiography (CTA) demonstrated occlusion of the left internal carotid artery, left vertebral artery, and left middle cerebral artery. Magnetic resonance imaging revealed an acute infarction in the left middle cerebral artery territory and the left lateral medulla with diffusion-fluid-attenuated inversion recovery (FLAIR) mismatch. Mechanical thrombectomy achieved successful recanalization of the left middle cerebral artery. However, severe cerebral edema developed the following day, requiring emergency decompressive craniectomy and intracranial pressure (ICP) monitoring. Intensive neurocritical care, including osmotherapy, deep sedation, controlled ventilation, and temperature management therapy guided by ICP monitoring, was performed. Cerebral edema gradually improved, and cranioplasty was performed on Day 44. Three months after injury, the patient had persistent right hemiparesis and aphasia with a modified Rankin Scale score of 5. This case highlights the importance of early recognition of BCVI in patients with cervical trauma, particularly when fractures involve the transverse foramen. Even when successful reperfusion is achieved, aggressive neurocritical care, including ICP monitoring and temperature management, may be essential for managing severe cerebral edema.

## Introduction

Blunt cerebrovascular injury (BCVI) is an uncommon but potentially devastating complication of blunt trauma involving the carotid or vertebral arteries. Although its reported incidence among trauma patients is low, BCVI is increasingly recognized with the widespread use of computed tomography angiography (CTA) in trauma screening [1,2]. Importantly, BCVI may cause delayed cerebral ischemia and stroke several hours to days after injury; therefore, early diagnosis and timely treatment are crucial for preventing secondary neurological deterioration [1,2].

Certain cervical fracture patterns, particularly upper cervical spine fractures and fractures involving the transverse foramen, are associated with an increased risk of BCVI [3,4]. When BCVI results in large-vessel occlusion and acute ischemic stroke, mechanical thrombectomy may be a reasonable treatment option in otherwise eligible patients [5]. However, even after successful recanalization, extensive infarction may lead to malignant cerebral edema requiring decompressive surgery and intensive neurocritical care, including intracranial pressure (ICP)-guided management [6,7]. Here, we report a case of traumatic internal carotid artery dissection associated with craniocervical junction trauma that resulted in middle cerebral artery occlusion, highlighting the importance of early recognition of BCVI and the challenges of post-reperfusion neurocritical care in severe ischemic stroke.

## Case presentation

A 57-year-old woman with a history of hypertension and prior lower-extremity fracture was transported after a fall. She had been taking amlodipine 5 mg/day and azilsartan 20 mg/day. She had no history of smoking or alcohol consumption and had been fully independent in her activities of daily living before the injury.

**Figure 1:**
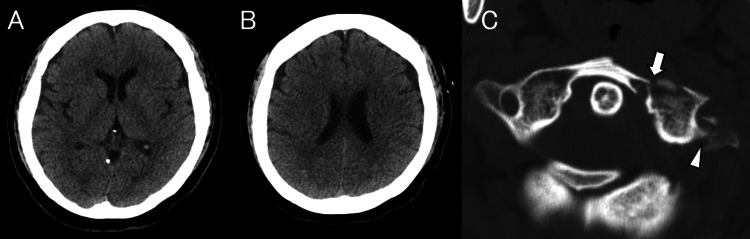
Initial head and cervical CT findings at the referring hospital. Non-contrast head CT demonstrating no skull fracture or intracranial hemorrhage (A, B). Cervical CT showing a fracture of the anterior arch of the atlas (white arrow) and a transverse process fracture extending to the left transverse foramen (arrowhead) (C).

**Figure 2:**
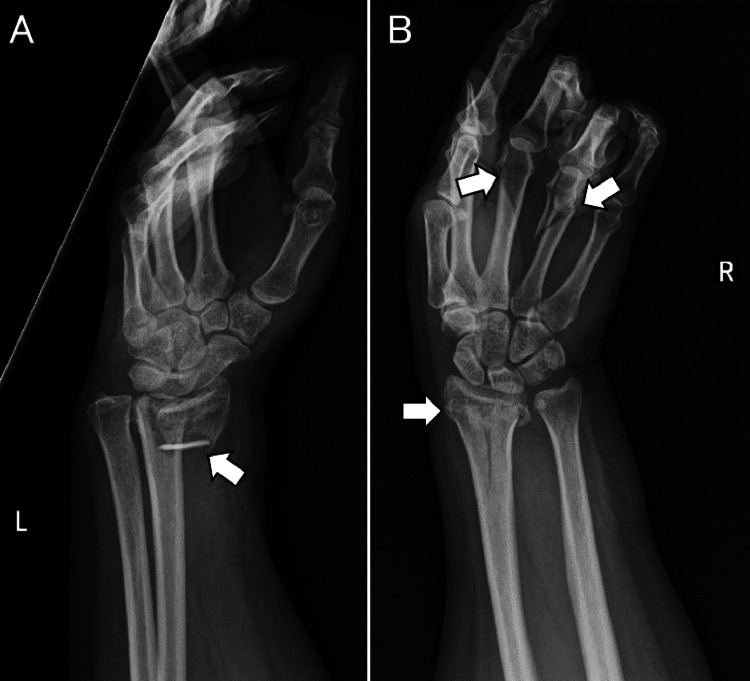
Plain radiographs of the upper extremities. Left wrist radiograph demonstrating a distal radius fracture (arrow) (A). Right hand and wrist radiograph demonstrating fractures of the third and fourth metacarpals and a distal radius fracture (arrows) (B).

On Day 0, she fell down six steps while carrying luggage at home and was transported to a referring hospital. On arrival, she was fully conscious, her vital signs were stable, and no limb paralysis was observed. Non-contrast head CT demonstrated no skull fracture or intracranial hemorrhage (Figure 1A, 1B), whereas cervical CT revealed fractures of the anterior and posterior arches of the atlas and a transverse process fracture extending to the left transverse foramen (Figure 1C). At that time, the patient was neurologically intact, and the initial management focused on trauma stabilization and treatment of associated orthopedic and soft-tissue injuries. Because no focal neurological deficits were present initially and urgent trauma management was prioritized, vascular imaging was not performed until neurological deterioration occurred. Plain radiographs demonstrated fractures of the right third and fourth metacarpals and bilateral distal radius fractures (Figure 2), for which splint fixation was applied by orthopedic surgeons. A scalp laceration with bone exposure was noted in the left temporal region and was sutured.

**Figure 3:**
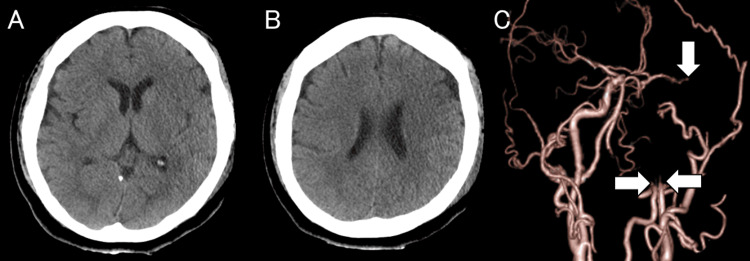
CT and three-dimensional CT angiography after neurological deterioration. Non-contrast head CT demonstrating subtle early ischemic changes in the left middle cerebral artery territory without intracranial hemorrhage (A, B). Three-dimensional CT angiography demonstrating occlusion of the left internal carotid artery, left vertebral artery, and left middle cerebral artery (arrows) (C).

Approximately four hours after the injury, she developed new right hemiparesis, aphasia, and left conjugate deviation. Repeat head CT showed no new intracranial hemorrhage but suggested subtle early ischemic changes in the left middle cerebral artery territory (Figure 3A, 3B). Because peripheral venous access was difficult due to severe obesity, vascular access had to be secured via a right femoral central venous catheter before contrast-enhanced imaging could be obtained. In parallel, trauma stabilization and treatment of associated orthopedic and soft-tissue injuries were ongoing. Contrast-enhanced CTA subsequently demonstrated poor opacification of the left internal carotid artery around the atlas fracture (Figure 3C), raising suspicion of traumatic cerebrovascular injury. Following this imaging assessment, inter-facility transfer to our comprehensive stroke center was arranged. As a result, approximately eight hours elapsed between injury and arrival at our institution.

She arrived at our hospital approximately eight hours after the injury. Her height was 160.0 cm, and her weight was 95.8 kg (body mass index, 37.4 kg/m²). She presented with severe disturbance of consciousness with a Glasgow Coma Scale (GCS) score of E1V1M1 (eye opening 1, verbal response 1, and motor response 1). Her blood pressure was 107/87 mmHg, heart rate 184 beats/min, respiratory rate 46 breaths/min, body temperature 36.6°C, and oxygen saturation 81% under a reservoir mask delivering 10 L/min oxygen. Because obstructive breathing was observed, a nasopharyngeal airway was inserted, which improved oxygen saturation to 95%.

Laboratory findings on admission are summarized in Table 1.

**Table 1:** Laboratory findings on admission. Laboratory data obtained on arrival at our hospital are shown. RBC: red blood cell count; AST: aspartate aminotransferase; ALT: alanine aminotransferase; ALP: alkaline phosphatase; pCO₂: partial pressure of carbon dioxide; pO₂: partial pressure of oxygen; LDL: low-density lipoprotein

Parameter	Result	Reference Range
RBC	4.60 ×10⁶ /μL	3.8-5.0 ×10⁶ /μL
Hemoglobin	13.2 g/dL	11.5-15.0 g/dL
Platelet count	26.7 ×10⁴ /μL	15-35 ×10⁴ /μL
Total protein	7.4 g/dL	6.5-8.0 g/dL
Total bilirubin	0.7 mg/dL	0.2-1.2 mg/dL
Blood urea nitrogen	12.7 mg/dL	8-20 mg/dL
Creatinine	0.39 mg/dL	0.5-1.0 mg/dL
Creatine kinase	134 U/L	40-200 U/L
AST	26 U/L	10-40 U/L
ALT	18 U/L	5-45 U/L
ALP	118 U/L	100-325 U/L
Prothrombin time	9.6 sec	10-13 sec
Activated partial thromboplastin time	25.9 sec	25-40 sec
D-dimer	30.2 μg/mL	<1.0 μg/mL
Sodium	134 mmol/L	135-145 mmol/L
Potassium	4.1 mmol/L	3.5-5.0 mmol/L
Total cholesterol	207 mg/dL	<200 mg/dL
LDL cholesterol	130 mg/dL	<120 mg/dL
Blood glucose	171 mg/dL	70-140 mg/dL
HbA1c	5.4%	4.6-6.2 %
pH	7.29	7.35-7.45
pCO₂	54 mmHg	35-45 mmHg
pO₂	114 mmHg	80-100 mmHg

Three-dimensional CTA performed at the referring hospital revealed occlusion of the left internal carotid artery, left vertebral artery, and distal M1 segment of the left middle cerebral artery (Figure 3C).

**Figure 4:**
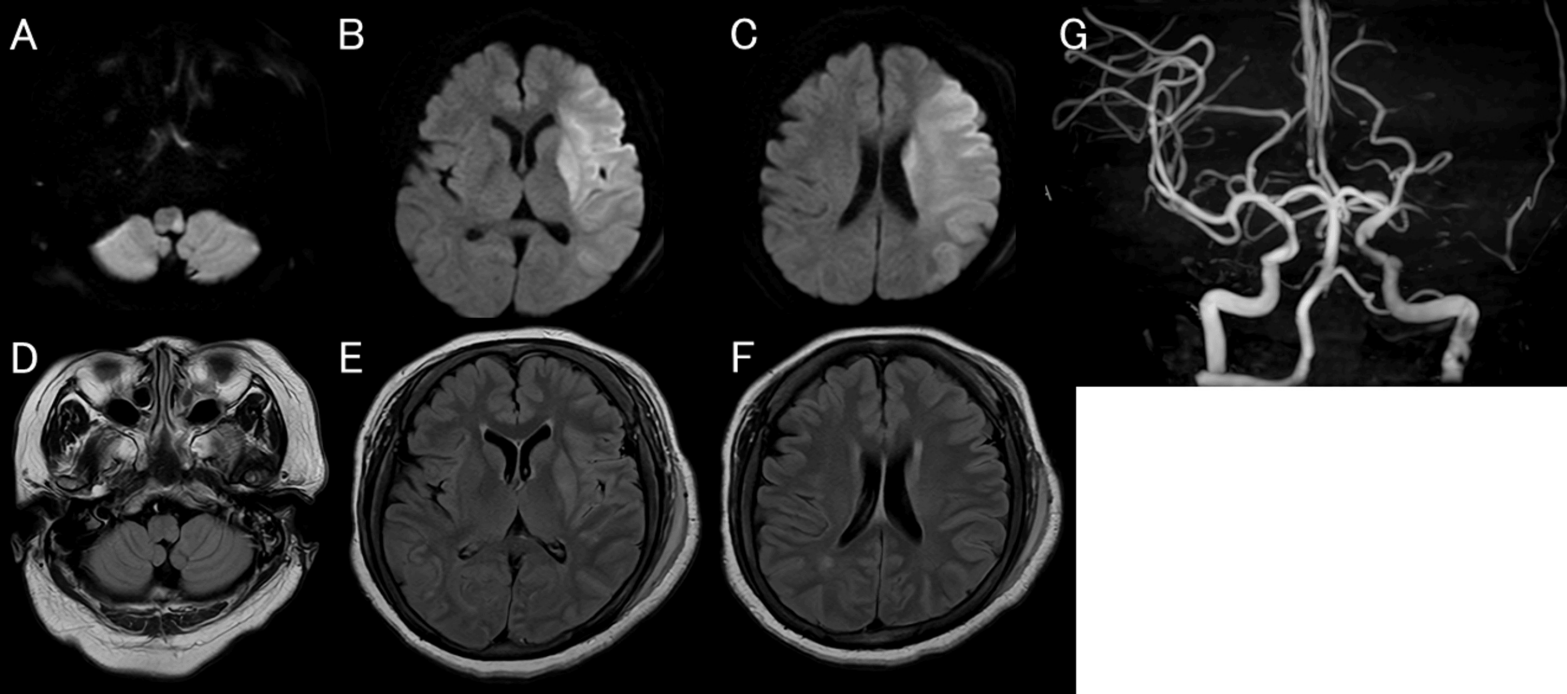
MRI and MRA findings on arrival at our hospital. Diffusion-weighted imaging (DWI) demonstrating acute infarction in the left lateral medulla and left middle cerebral artery territory (A-C). Fluid-attenuated inversion recovery (FLAIR) showing no corresponding signal change (D-F), indicating a DWI-FLAIR mismatch. Magnetic resonance angiography demonstrating occlusion of the left vertebral artery and left middle cerebral artery, with a signal defect in the left internal carotid artery (G).

An MRI performed after arrival at our hospital demonstrated acute infarction in the left middle cerebral artery territory, with a diffusion-weighted imaging Alberta Stroke Program Early CT Score (DWI-ASPECTS) of 2. Additional acute infarction was also seen in the left lateral medulla (Figure 4A-4C). Fluid-attenuated inversion recovery (FLAIR) images showed no corresponding signal change (Figure 4D-4F), indicating a DWI-FLAIR mismatch and supporting the presence of salvageable tissue despite subtle early ischemic changes on the preceding CT. Magnetic resonance angiography (MRA) demonstrated left vertebral artery occlusion, a signal defect in the left internal carotid artery, and left middle cerebral artery occlusion (Figure 4G). Although the ischemic lesion was already extensive, the absence of corresponding FLAIR hyperintensity suggested hyperacute infarction with possible residual salvageable tissue. Given the severe neurological deficit and persistent large-vessel occlusion, mechanical thrombectomy was considered a reasonable treatment option.

**Figure 5:**
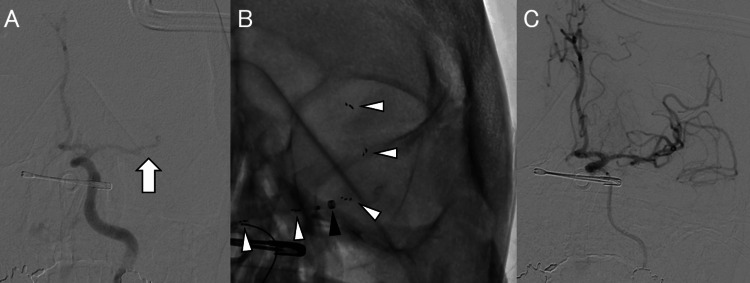
Mechanical thrombectomy procedure. Initial angiography demonstrating occlusion of the left middle cerebral artery (arrow) (A). A stent retriever (white arrowhead) was deployed across the thrombus while an aspiration catheter (black arrowhead) was advanced into the clot (B). Final angiography demonstrating successful recanalization of the left middle cerebral artery (C).

Subsequent cerebral angiography revealed findings suspicious for dissection of the left internal carotid artery at the C1 level near the craniocervical junction, with residual antegrade flow. Occlusion of the left middle cerebral artery and left vertebral artery was also confirmed (Figure 5A).

The patient was diagnosed with left middle cerebral artery occlusion due to traumatic left internal carotid artery dissection. Mechanical thrombectomy was therefore performed. An 8-Fr guiding catheter (Optimo®, Tokai Medical Products, Aichi, Japan) was placed in the left common carotid artery via a right femoral artery approach. An aspiration catheter (Catalyst 6®, Stryker Neurovascular, Fremont, CA, USA), microcatheter (Phenom 21®, Medtronic, Irvine, CA, USA), and microguidewire (CHIKAI Nexus®, Asahi Intecc, Aichi, Japan) were advanced to the occlusion site. A stent retriever (Solitaire X 4×40 mm®, Medtronic, Irvine, CA, USA) was deployed across the thrombus while aspiration was applied (Figure 5B). Successful recanalization of the left middle cerebral artery (TICI 2B) was achieved with a single pass (Figure 5C). The door-to-picture time was 18 minutes, door-to-puncture time 68 minutes, and door-to-revascularization time 83 minutes.

**Figure 6:**
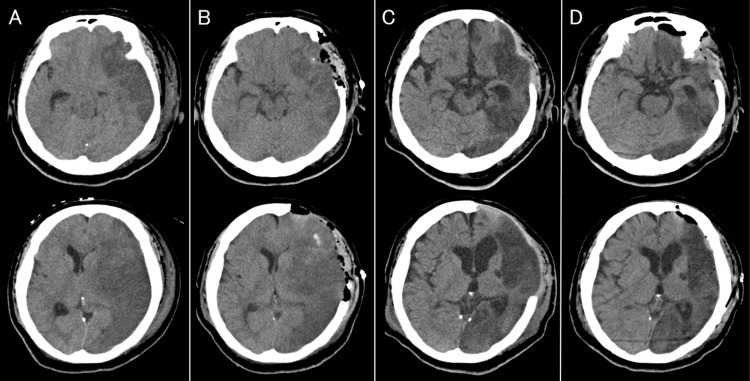
Serial changes on head CT. Day 1 CT demonstrating extensive infarction in the left middle cerebral artery territory with midline shift and uncal herniation (A). CT after decompressive craniectomy on Day 2 showing improvement of midline shift and resolution of uncal herniation (B). CT on Day 40 demonstrating resolution of cerebral edema (C). CT after cranioplasty on Day 44 (D).

Despite successful reperfusion, the patient remained unconscious and required airway protection; therefore, endotracheal intubation and mechanical ventilation were initiated. On Day 1, anisocoria (right pupil 2 mm, left pupil 5 mm) and loss of the pupillary light reflex were observed, suggesting impending brain herniation due to progressive cerebral edema. Head CT revealed worsening swelling of the left middle cerebral artery infarction with mild brainstem compression (Figure 6A). Emergency decompressive craniectomy and ICP monitoring were therefore performed (Figure 6B).

Postoperatively, intensive neurocritical care was initiated, including strict blood pressure control with a target systolic blood pressure of 110-140 mmHg, head elevation to 30°, osmotherapy with mannitol and glycerol, deep sedation with midazolam and propofol, temperature management using an intravascular cooling catheter (Cool Line®, ZOLL Medical, Chelmsford, MA, USA) with an initial target temperature of 36°C, and ventilatory management targeting an end-tidal CO₂ of 35 mmHg. On Day 2, ICP exceeded 20 mmHg; therefore, the target end-tidal CO₂ was adjusted from 35 mmHg to 30 mmHg, and active temperature management was continued. On Day 4, ICP increased to 25 mmHg, although the perimesencephalic cisterns remained preserved on CT. The target temperature was then lowered from 36°C to 35°C, after which the pupillary findings gradually improved. By Day 8, ICP had stabilized below 20 mmHg, and CT demonstrated improvement of the herniation findings. Rewarming was initiated at a rate of 1°C/day, and sedation was gradually tapered without rebound ICP elevation. Because prolonged ventilatory support was anticipated, tracheostomy was performed on Day 9. Cerebral edema gradually improved thereafter (Figure 6C). Cranioplasty was performed on Day 44 (Figure 6D). CTA performed after stabilization demonstrated persistent left internal carotid artery dissection and stenosis. After decompressive craniectomy and subsequent cranioplasty, aspirin was initiated for secondary stroke prevention once the immediate postoperative hemorrhagic risk was considered acceptable.

The atlas fracture was managed conservatively with a cervical collar, which was removed after confirmation of bone healing.

Approximately two months after the injury, the patient had a GCS score of E4VTM6 (eye opening 4, verbal response T (tracheostomy), motor response 6) with persistent right hemiparesis and aphasia. No further neurological deterioration occurred during hospitalization. At three months after the injury, her modified Rankin Scale score was 5.

## Discussion

This case involved traumatic left internal carotid artery dissection and left vertebral artery occlusion associated with craniocervical junction trauma, resulting in left middle cerebral artery occlusion. Although mechanical thrombectomy achieved reperfusion, malignant cerebral edema subsequently progressed, requiring decompressive craniectomy and intensive neurocritical care. The clinical significance of this case lies in two points: the importance of early recognition of BCVI after craniocervical trauma and the critical role of neurocritical care after reperfusion for malignant cerebral edema.

BCVI is an uncommon vascular injury after blunt trauma, but it can cause devastating complications, including ischemic stroke and death, and the outcome is strongly influenced by early diagnosis and appropriate treatment [1]. Cerebral ischemia caused by BCVI often develops several hours to days after injury, and the diagnosis may therefore be missed when the patient has no obvious neurological deficits during the initial trauma assessment [1,2]. In the present case, the patient was neurologically intact immediately after the fall, but she developed right hemiparesis and aphasia several hours later, at which point severe cerebral ischemia became clinically apparent. This temporal profile underscores the need to suspect vascular injury on the basis of injury mechanism and fracture pattern, even when initial neurological findings are limited.

Recent studies have shown that upper cervical fractures (occiput-C3), multilevel fractures, fracture-dislocations or subluxations, and fractures involving the transverse foramen are associated with BCVI [3,4]. In such fracture patterns, CTA-based vascular screening is recommended because early diagnosis may reduce the risk of subsequent stroke [2]. In the present case, the C1 fracture with extension into the transverse foramen represented a high-risk fracture pattern for BCVI and would meet commonly used screening indications such as the expanded Denver criteria [1,2]. In retrospect, immediate CTA at the time of initial trauma evaluation may have enabled earlier diagnosis. However, because the patient was neurologically intact at first presentation and the initial management appropriately focused on trauma stabilization and treatment of associated injuries, the vascular injury was not recognized until neurological deterioration occurred. This case highlights an important practical challenge in BCVI screening: high-risk injury patterns must prompt vascular evaluation even in patients who initially appear neurologically stable.

Another important lesson from this case is that delays may occur even after neurological deterioration becomes clinically apparent. In trauma settings, the pathway to vascular diagnosis and stroke treatment may be prolonged by competing priorities such as stabilization of associated injuries, technical difficulties in obtaining contrast-enhanced imaging, and inter-facility transfer logistics. In the present case, these real-world factors likely contributed to the interval between symptom onset and arrival at our hospital. This underscores that “early recognition” of BCVI requires not only awareness of high-risk injury patterns, but also rapid execution of vascular imaging and transfer pathways once neurological deterioration occurs.

This patient had multiple-vessel BCVI, with both left internal carotid artery dissection and left vertebral artery occlusion. Recent studies suggest that patients with injuries involving multiple cervical vessels are at greater risk of radiographic progression, in-hospital stroke, and death than those with a single-vessel injury [8,9]. Hosseinpour et al. reported that multiple-vessel BCVI was associated with a significantly higher frequency of lesion progression and stroke on follow-up imaging [8]. Wagner et al. further suggested that the combination of carotid and vertebral artery injuries may itself increase stroke risk [9]. These findings support the need for serial vascular imaging in patients such as ours, in whom more than one cervical vessel is affected.

In the acute phase, mechanical thrombectomy achieved successful reperfusion (TICI 2B) for middle cerebral artery occlusion caused by traumatic cervical artery dissection. According to the American Heart Association scientific statement on cervical artery dissection, mechanical thrombectomy is reasonable in otherwise eligible patients with acute ischemic stroke due to cervical artery dissection [5]. In addition, a systematic review and meta-analysis of dissection-related tandem occlusions suggested that endovascular treatment is feasible, with acceptable safety and high recanalization rates [10]. In the present case, the indication for thrombectomy was challenging because the DWI-ASPECTS was already 2, suggesting a large ischemic core and a high risk of a poor functional outcome. However, the patient was relatively young, had a devastating neurological presentation, and remained within a hyperacute time window. Moreover, the absence of corresponding FLAIR hyperintensity suggested DWI-FLAIR mismatch, raising the possibility that some tissue remained salvageable despite the low ASPECTS. On this basis, thrombectomy was considered a reasonable rescue strategy. However, our patient subsequently developed severe cerebral edema despite successful recanalization. This case, therefore, illustrates that successful reperfusion does not necessarily translate into a favorable functional outcome when infarction is already extensive.

An additional clinically important feature of this case was the concurrent acute infarction of the left lateral medulla associated with left vertebral artery occlusion. Although the malignant cerebral edema caused by the large left middle cerebral artery infarction was the dominant life-threatening pathology, the brainstem infarction may also have significantly influenced the patient’s presentation and recovery. The lateral medulla contains structures involved in autonomic regulation, respiratory pattern generation, swallowing, and airway protection. Therefore, the marked tachycardia and tachypnea observed on arrival, as well as the prolonged need for airway support and tracheostomy, may have reflected not only the effects of hemispheric infarction and mass effect, but also the contribution of the concomitant medullary infarction. The coexistence of supratentorial and brainstem ischemia likely increased the overall neurological burden and may have contributed to the poor functional outcome.

A key feature of this case was the role of neurocritical care after reperfusion. After anisocoria emerged, emergency decompressive craniectomy and ICP monitoring were performed, followed by multimodal management, including head elevation, osmotherapy, deep sedation, ventilatory control, and temperature management. The Neurocritical Care Society guidelines support hyperosmolar therapy and ICP monitoring in the management of cerebral edema and elevated ICP [6]. By contrast, routine therapeutic hypothermia after decompressive surgery for malignant middle cerebral artery infarction has not been shown to improve functional outcome and is not recommended as standard care [7]. In the present case, body temperature was adjusted under continuous ICP monitoring, and a lower temperature target was selected when ICP rose, allowing ICP-guided temperature titration rather than fixed therapeutic hypothermia. Fever itself is also associated with worse stroke outcomes, and fever prevention remains an important component of neurocritical care [11,12]. Taken together, this case suggests that, even after recanalization, patients with extensive infarction due to traumatic BCVI may require aggressive neurocritical care, including ICP monitoring and careful temperature management.

Another important management issue in this case was the choice of secondary antithrombotic therapy for traumatic cervical artery dissection involving both the carotid and vertebral circulations. Although both antiplatelet therapy and anticoagulation are used in cervical artery dissection, current evidence does not clearly support the universal superiority of one approach over the other. In the present case, aspirin was selected after the acute neurosurgical phase because the patient had already developed a large infarction, had undergone decompressive craniectomy, and was considered to be at substantial risk for intracranial hemorrhagic complications. Under these circumstances, antiplatelet therapy was considered a more cautious and practical strategy than anticoagulation.

From a prognostic standpoint, the unfavorable outcome in this case was likely shaped by two closely related factors: delayed recognition of BCVI and intervention after a large ischemic core had already developed. In addition, because follow-up vascular imaging was not obtained after thrombectomy, the exact mechanism of the subsequent deterioration cannot be definitively determined. Re-occlusion, infarct progression despite reperfusion, and reperfusion-related injury cannot be fully distinguished. Therefore, the poor outcome should be interpreted not simply as a reflection of disease severity alone, but as the combined result of delayed diagnosis, advanced infarction at the time of treatment, and limited certainty regarding the exact postprocedural vascular course.

## Conclusions

This case highlights the diagnostic and management challenges of BCVI after cervical trauma, particularly when delayed neurological deterioration occurs in the setting of multi-vessel injury. It also illustrates that, even after technically successful reperfusion, patients with extensive infarction may require aggressive neurocritical care for malignant cerebral edema. Although conclusions from a single case must remain cautious, this report underscores the importance of maintaining suspicion for BCVI, expediting vascular evaluation and transfer, and integrating endovascular and neurocritical care strategies when severe ischemic complications occur.
